# Efficacy and Safety of Exploring Deeper Sections of the Infrapapillary Area of the Duodenum by Using Sedative Esophagogastroduodenoscopy

**DOI:** 10.1155/2022/1381299

**Published:** 2022-07-27

**Authors:** Ming-Tse Hsu, Chi-Yi Chen, Kai-Sheng Liao, Wei-Sheng Chung

**Affiliations:** ^1^Division of Gastroenterology, Department of Internal Medicine, Ditmanson Medical Foundation, Chiayi Christian Hospital, Chiayi, Taiwan; ^2^Department of Pathology, Ditmanson Medical Foundation, Chiayi Christian Hospital, Chiayi, Taiwan; ^3^Department of Internal Medicine, Taichung Hospital, Ministry of Health and Welfare, Taichung, Taiwan; ^4^Department of Health Services Administration, China Medical University, Taichung, Taiwan; ^5^Department of Healthcare Administration, Central Taiwan University of Science and Technology, Taichung, Taiwan

## Abstract

**Background:**

Using conventional esophagogastroduodenoscopy (EGD) to evaluate the infrapapillary area is not feasible. The use of sedative EGD may enable endoscopists to investigate the infrapapillary condition of the duodenum. In this study, we aimed to evaluate lesions in the infrapapillary regions by using sedative EGD.

**Methods:**

In this retrospective observational study, we used the data of patients who underwent sedative EGD examinations at a tertiary hospital in southern Taiwan. The endoscopists evaluated the esophagus, stomach, and proximal duodenum and then attempted to explore the infrapapillary portion of the duodenum as deeply as possible. We assessed the success rate for the exploration of infrapapillary areas. Furthermore, we analyzed specific clinical findings of sedative EGD examination.

**Results:**

In total, 2973 patients underwent sedative EGD between November 1, 2010, and December 31, 2011. For 2632 of these patients, it was their first sedative EGD examination. In 2511 patients (95.4%), the exploration of the infrapapillary areas was successful. In approximately 10% of the patients, specific findings were detected over the infrapapillary region, and 7 of these patients exhibited clinically significant findings (i.e., gallbladder cancer with metastasis, periampullary Vater adenoma, natural killer cell enteropathy, villous adenoma with moderate dysplasia, infrapapillary duodenal adenoma with dysplasia, duodenal perforation with tumor-like formation, and follicular lymphoma). No patient experienced minor or major adverse reactions during the sedative EGD procedure.

**Conclusions:**

The current study provided evidence that sedative EGD examination enables a safe, comfortable, and effective endoscopic examination of deeper sections of the duodenum to evaluate the papillary and infrapapillary regions.

## 1. Introduction

Esophagogastroduodenoscopy (EGD) is widely applied for examining the esophagus, stomach, and duodenum. In the past, many endoscopists in Taiwan did not use sedation when conducting conventional EGD to evaluate patients. Because of the discomfort resulting from this procedure, endoscopists typically pass the fiberoptic endoscope rapidly through the mouth or nose into the oropharynx, esophagus, stomach, and proximal duodenum after applying topical oral anesthesia. Most duodenal lesions, especially those related to duodenal ulcers, are localized in the bulb. However, the rate of detection for duodenal polyps and tumors in patients when using gastroscopic examination is relatively low [1]. A related study indicated that duodenal tumors comprise 35% of all benign and 17% of malignant small bowel neoplasia [2]. The majority of duodenal cancers arise in the second portion of the duodenum, followed by cancers in the papillary and infrapapillary areas of the duodenum, which are also aggressive [3]. The incidence of ampullary tumors has increased because of the widespread use of routine EGD surveillance for health examination [4]. Early-stage ampullary cancers limited to the ampulla of Vater have 5-year survival rates of 80% to 100% [5, 6]. Computed tomography (CT) and magnetic resonance imaging modalities with intravenous contrast administration are useful because they enable a comprehensive evaluation of the duodenum [7]; however, EGD enables endoscopic biopsy for pathological diagnosis without serious complications [8].

The American Society of Gastrointestinal Endoscopy (ASGE) and relevant studies have not addressed and defined the area of the second portion of the duodenum by using EGD. Moreover, the use of conventional EGD to evaluate the infrapapillary area was not feasible in the past and resulted in patient discomfort. The objectives of using sedatives during EGD are to alleviate patient discomfort and improve the results of the technical examination. Therefore, sedative EGD has become popular because it enables endoscopists to approach and examine the second portion of the duodenum and more easily proceed deeper into the infrapapillary area while maximizing patient comfort. In addition to following ASGE guidelines for the standard of upper endoscopy, we attempted to evaluate the papillary and infrapapillary regions by using sedative EGD. The aim of this study was to evaluate lesions around the papillary and infrapapillary regions by using sedative EGD. Modification of EGD guidelines should be considered when deliberating the cost and benefit of sedative EGD.

## 2. Methods

### 2.1. Patient and Public Involvement

A retrospective observational study was undertaken on patients receiving sedative EGD examination from November 1, 2010, to December 31, 2011, in Chiayi Christian Hospital (Chiayi City, southern Taiwan). The hospital has 1077 acute beds and serves approximately 4110 outpatients. The current data were obtained from both inpatients and outpatients who underwent EGD for clinical and health surveillance.

### 2.2. Procedure

All the participants signed informed consent forms for the EGD procedure and sedation. The EGD examinations were conducted by using the Olympus end-viewing video endoscope (GIF-Q260 and GIF-H260Z, Aizu Olympus Co., Ltd., Japan). Before the sedative EGD procedure, all patients received anesthetic consultations and risk evaluation. We implemented sedative EGD examination for the patients whose anesthesia evaluation was categorized as levels I, II, and III according to the American Society of Anesthesiologists physical status classification [9]. Moreover, an anesthesiologist accompanied the endoscopist during the EGD procedure. Propofol was the most common sedative used. In contrast, we compared 100 cases to be the control group. Those patients performed nonsedative EGD in the control group. Before the EGD examination, the patients received a dimethicone solution, a lidocaine jelly, and a hyoscine N-butylbromide intramuscular injection in the absence of contraindication. We excluded the patients with prior history of gastric surgery or gastric outlet obstruction. All endoscopists visualized the esophagus, stomach, and proximal duodenum; subsequently, they attempted to explore the infrapapillary portion of the duodenum as deeply as possible. If lesions were detected during the EGD examinations, the endoscopist had the option of taking a tissue biopsy for pathological examination. Endoscopic diagnoses around the papillary and infrapapillary portions of the duodenum were either considered to be clinically significant (precancer, cancer, or solitary bleeder) or insignificant. This study was approved by the Institutional Review Board of Chiayi Christian Hospital (IRB 2019064). Informed consent was waived because of the deidentification of patient data.

## 3. Results

In total, 2973 patients underwent sedative EGD during the study period. For 2632 of these patients, the sedative EGD examination at Chiayi Christian Hospital was their first. The majority of these patients were female (55.4%), and the average age was 53.2 ± 14.4 years. In contrast, the mean age of the patients who received EGD without sedation was 58.4 ± 16.4 years. Most of the indications for EGD examination were upper abdominal symptoms (65.5% for sedative EGD and 56% for EGD without sedation). After EGD examination, we followed up with these patients one week later and none of the patients had an emergent cardiopulmonary insult. In addition, the patients with sedative EGD examination did not feel discomfort in their memory during follow-up one week later (Table 1).

**Figure 1:**
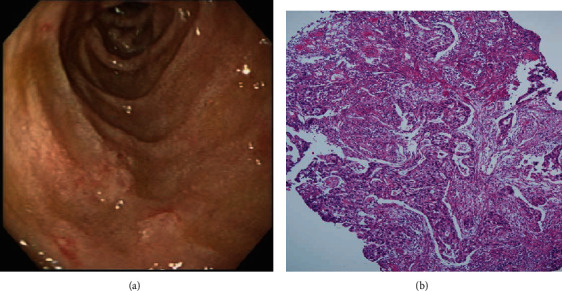
(a) Small polypoid lesions with shallow ulcers over the infrapapillary area (arrow). (b) Duodenal involvement of adenocarcinoma, featuring neoplastic epithelial cells arranged in irregular glands infiltrating the stroma (H&E, 100x).

**Figure 2:**
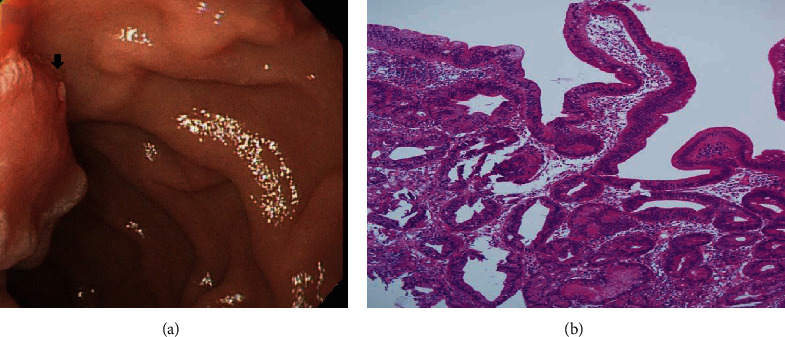
(a) Flat periampullary Vater tumor (arrow). (b) Duodenal tubular adenoma with mild dysplasia composed of dysplastic glandular cells with low-grade dysplasia in tubular architecture (H&E, 100x).

**Figure 3:**
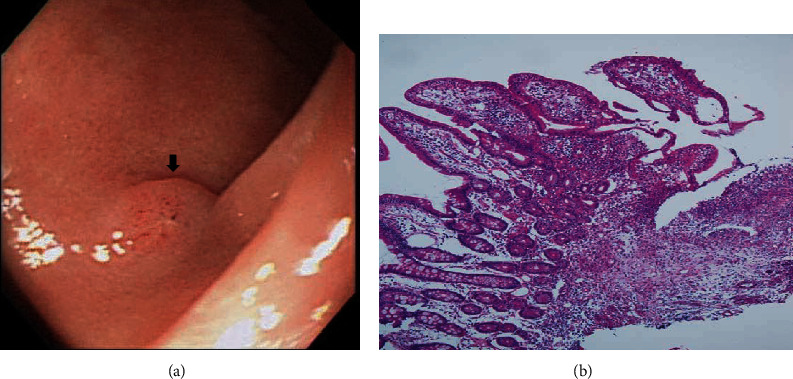
(a) Shallow ulcer over the infrapapillary area (arrow). (b) NK cell enteropathy composed of mononuclear cells infiltrating the duodenal mucosa. Further immunohistochemical staining confirmed the NK cell phenotype (CD3+/CD56+/TIA-1+) (H&E, 100x).

**Figure 4:**
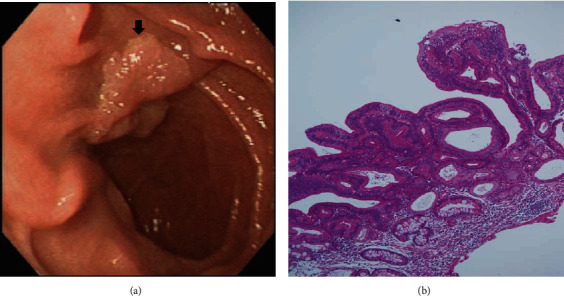
(a) Flat polyp over the infrapapillary area (arrow). (b) Duodenal villous adenoma with moderate dysplasia composed of dysplastic glandular cells with low-grade dysplasia in villous architecture (H&E, 100x).

**Figure 5:**
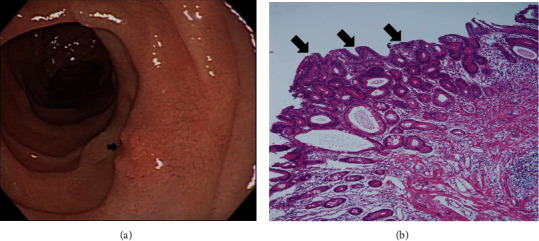
(a) Ulcerated lesion over the infrapapillary area (arrow). (b) Mucosal glands of the duodenum with focal low-grade dysplasia (arrow) (H&E, 100x).

**Figure 6:**
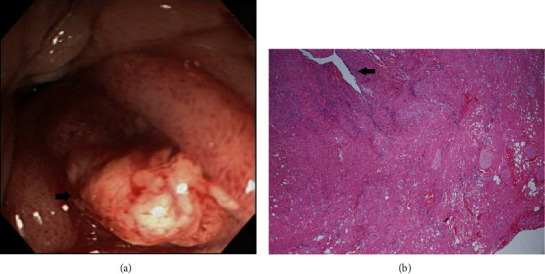
(a) Collapsed lumen with ulcerated mass obstruction over the infrapapillary area (arrow). (b) Transmural fistular tract (arrow) in the third portion of the duodenum with perforation and inflammation (H&E, 40x).

**Figure 7:**
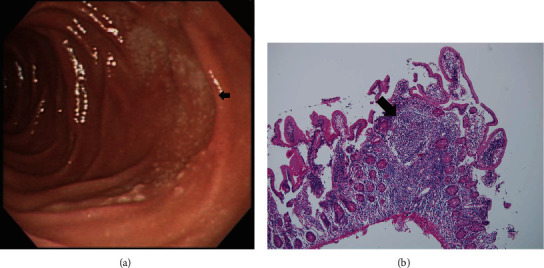
(a) White patches and nodules over the infrapapillary area (arrow). (b) Duodenal follicular lymphoma (arrow), featuring multiple lymphoid follicles in the lamina propria, composed of relatively uniform small lymphoid cells. Further immunohistochemical staining confirmed follicular lymphoma (CD20+/BCL2+/BCL6+/CD10+) (H&E, 100x).

On the basis of whether the exploration of the papillary and infrapapillary areas was successful, we further divided the patients into 2 groups: a success group (*n* = 2511, 95.4%) and a failure group (*n* = 121, 4.6%). In the failure group, 14 patients had prior gastroduodenal resection with bowel anastomosis; in the remaining 107 patients, the examination of the papillary and infrapapillary regions failed because of local anatomic changes (10 patients), a history of abdominal surgery with suspicious adhesion (12 patients), the effect of an intra-abdominal mass (6 patients), and an intractable technical approach (79 patients). In the success group, 2260 patients had negative findings over the papillary and infrapapillary areas. However, we detected specific findings over these regions in 251 patients. Furthermore, we found clinically significant lesions such as cancer, precancerous lesions (adenoma and dysplasia), and solitary bleeders in 7 patients. The remaining 244 patients had clinically insignificant findings (e.g., lymphangiectasia, hyperplasia, inflammatory polyps, and duodenitis). Next, we described the 7 patients with clinically significant lesions over the papillary or infrapapillary regions. The first patient was a 58-year-old woman complaining of epigastralgia. The sedative EGD revealed a duodenal polyp and nodule, and the biopsy indicated adenocarcinoma composed of moderately differentiated neoplastic glands infiltrating the stroma (Figures 1(a) and 1(b)). The final diagnosis for this patient was gallbladder cancer with metastasis. The second patient was a 61-year-old woman complaining of epigastralgia. The examination revealed a periampullary Vater tumor, and the subsequent biopsy indicated tubular adenoma, featuring low-grade dysplasia of the mucosal glands (Figures 2(a) and 2(b)). The final diagnosis for this patient was periampullary Vater adenoma. The third patient was a 63-year-old woman complaining of epigastralgia. The examination revealed a duodenal ulcer, and the subsequent biopsy indicated natural killer (NK) cell enteropathy composed of CD56+ mononuclear cell infiltrates in the duodenal mucosa (Figures 3(a) and 3(b)). The fourth patient was a 55-year-old man reporting positive occult blood in feces. The examination revealed a duodenal polyp, and the subsequent biopsy indicated villous adenoma with moderate dysplasia (Figures 4(a) and 4(b)). The fifth patient was a 54-year-old man undergoing a health examination. The examination revealed a duodenal ulcer, and the biopsy pathology indicated low-grade glandular dysplasia (Figures 5(a) and 5(b)). The final diagnosis for this patient was infrapapillary duodenal adenoma with low-grade dysplasia. The sixth patient was a 58-year-old man complaining of tarry stool. The examination revealed a duodenal tumor in the third portion, and the resected specimen exhibited a transmural fistula tract with perforation, possibly due to mechanical injury resulting from a fish bone or perforated diverticulum (Figures 6(a) and 6(b)). The seventh patient was a 65-year-old woman complaining of epigastralgia. The examination revealed duodenal white patches in the second portion, and the biopsy pathology indicated follicular lymphoma, featuring multiple lymphoid follicles composed of centrocyte-like lymphoid cells in the lamina propria (Figures 7(a) and 7(b)). We conducted EGD in the control group following guidelines to the 2nd portion and further pushed the endoscope as deeper as possible but in vain due to discomfort and intolerance to the procedure.

## 4. Discussion

The results indicated that the sedative EGD examination reached the papillary and infrapapillary regions in 2511 of the 2632 patients (95.4%). Furthermore, specific findings were detected over the infrapapillary region in 10% of these patients, with 7 patients exhibiting clinically significant findings. Sedative EGD enables a safe, comfortable, and effective endoscopic examination, which facilitates the investigation of the second portion of the duodenum and even downward to the infrapapillary region [10–13]. When endoscopists conduct nonsedative EGD examinations to evaluate the oropharynx, esophagus, stomach, and proximal duodenum with real-time assessment, patients may experience irritation due to air insufflation in the stomach and discomfort from gagging during the procedure [14]. Therefore, the evaluation of the major duodenal papilla by using conventional nonsedative EGD is not feasible [15]. A side-viewing endoscopy enables a clearer view of the major duodenal papilla. Nonetheless, the current side-viewing endoscopy is not appropriate for routine screening in EGD examination [16].

Previous studies have indicated that the diagnostic yield of endoscopy for appropriate ASGE indications was higher than that for inappropriate ASGE indications [17, 18]. ASGE guidelines and indications have not clarified the depth of EGD for duodenal examinations, which may result in practitioners missing clinically relevant diagnoses of the duodenal papilla in the infrapapillary region. Our sedative EGD examinations resulted in specific findings over the infrapapillary region in approximately 10% of patients; among them, 7 patients exhibited clinically significant findings. The presence of lesions in the second portion of the duodenum is uncommon, accounting for approximately 38% of small bowel tumors located in the duodenum. Lesions in the peripapillary region are more common than those in the suprapapillary and infrapapillary sites. In our study, 6 patients had malignant and premalignant diagnoses, and 1 patient presented with duodenal perforation, including tumor formation and bleeding. The EGD examination of the first patient revealed ulcers over the infrapapillary region, and the biopsy revealed adenocarcinoma compatible with invasion from gallbladder cancer. Related reports have indicated that patients with gallbladder papillary adenocarcinoma have a more favorable prognosis than those with nonpapillary gallbladder carcinomas [19, 20]. This may be attributed to the relatively delayed invasion of gallbladder papillary adenocarcinoma into the gallbladder wall.

The EGD examination of the seventh patient revealed white patches over the infrapapillary region of the duodenum, and the subsequent biopsy revealed follicular lymphoma. Follicular lymphoma most commonly presents with widespread lymphadenopathy and is less frequently observed in the gastrointestinal tract [21]. Duodenal-type follicular lymphoma, identified by its restriction to the duodenum, is a rare variant of follicular lymphoma [22]. Relative to other types of follicular lymphoma, duodenal-type follicular lymphoma tends to remain at a low stage and has a low frequency of progression and dissemination [23, 24].

The EGD examination of the third patient revealed small superficial ulcers over the infrapapillary region of the duodenum, and the biopsy revealed mucosal infiltrates of mononuclear cells with a positive NK cell phenotype (CD3+/CD45+/CD56+/TIA-1+), which was consistent with NK cell enteropathy. The Epstein–Barr encoding region (EBER) in *in situ* hybridization was negative [25]. The Epstein–Barr virus is widely regarded as a culprit in malignant stimulation. In *in situ* hybridization, the EBER may become positive in NK cell lymphoma [26]. NK cell enteropathy is a lymphoproliferative disorder rather than NK cell lymphoma in the gastrointestinal tract and may present a relatively indolent clinical course [27]. The real cause of NK cell enteropathy remains to be elucidated.

The EGD examination of the second patient disclosed a periampullary Vater tumor, and the subsequent biopsy indicated tubular adenoma, featuring low-grade dysplasia of the mucosal glands. The European Society of Gastrointestinal Endoscopy Guideline for the endoscopic management of ampullary tumors recommends endoscopic papillectomy for patients with periampullary Vater adenoma without intraductal extension or *en bloc* resection for lesions up to 20-30 mm in diameter [28]. Our patient with periampullary Vater adenoma had a history of end-stage renal disease and hesitated for endoscopic or surgical intervention risk. Fortunately, no interval change was present after a 10-year follow-up endoscopy.

The EGD examination of the sixth patient revealed a bleeder over the infrapapillary region of the duodenum. Abdominal CT and exploratory laparotomy confirmed duodenal perforation with tumor-like formation and bleeding. Although only a few patients in this study exhibited clinically significant lesions over the papillary and infrapapillary regions, endoscopists should consider deeper endoscopic examinations by using sedative EGD. The duodenoscope in endoscopic retrograde cholangiopancreatography (ERCP) and enteroscopy still play a major role in the evaluation of lesions over the papillary and infrapapillary regions. However, ERCP and enteroscopy are time-consuming, require patient cooperation, and have additional risks [29, 30].

The current results indicated that the sedative EGD examination is safe and effective for the evaluation of lesions over the papillary and infrapapillary regions. No patient developed perforation or bleeding as a result of the EGD procedure, nor did any patient experience cardiopulmonary events during the procedure. However, several limitations must be considered when interpreting the current findings. First, the current study did not conduct a randomized control trial to compare sedative EGD examination and nonsedative EGD examination. Second, the study period seemed relatively old.

## 5. Conclusions

The current study provided evidence that sedative EGD examination helps endoscopists examine deeper sections of the duodenum to safely and easily evaluate the papillary and infrapapillary regions.

## Figures and Tables

**Table 1:** Demographic and clinical characteristics between patients receiving EGD with and without sedation.

Variables	Sedative EGD *N* (%)	EGD without sedation *N* (%)
Sex		
Male	1174 (44.6)	51 (51)
Female	1458 (55.4)	49 (49)
Age (y)		
Mean ± SD	53.2 ± 14.4	58.4 ± 16.4
Indication for EGD		
Health examination	602 (22.9)	2 (2)
Upper abdominal symptoms	1724 (65.5)	56 (56)
Gastrointestinal bleeding	172 (6.5)	8 (8)
Follow-up of previous ulcer and cancer	134 (5.1)	34 (34)
Successful exploration of the papillary and infrapapillary areas	2511 (95.4)	0 (0)
Cardiopulmonary events during examination	0 (0)	0 (0)

EGD: esophagogastroduodenoscopy; SD: standard deviation.

## Data Availability

All data generated and analyzed in this study were included in this published article.

## Abbreviations

EGD: Esophagogastroduodenoscopy
ASGE: American Society of Gastrointestinal Endoscopy
IRB: Institutional review board
NK: Natural killer
EBER: Epstein–Barr encoding region
CT: Computed tomography
ERCP: Endoscopic retrograde cholangiopancreatography.

## Additional Points

*Callout Box*. (i) What is known on this topic? Esophagogastroduodenoscopy (EGD) is widely applied for examining the esophagus, stomach, and duodenum. In the past, many endoscopists in Taiwan did not use sedation when conducting conventional EGD to evaluate patients. (ii) What this study adds. The current study provided evidence that sedative EGD examination enables a safe, comfortable, and effective endoscopic examination of deeper sections of the duodenum to evaluate the papillary and infrapapillary regions.
